# Revealing the secret life of skin ‐ with the microbiome you never walk alone

**DOI:** 10.1111/ics.12594

**Published:** 2019-12-25

**Authors:** R. Sfriso, M. Egert, M. Gempeler, R. Voegeli, R. Campiche

**Affiliations:** ^1^ DSM Nutritional Products Personal care Wurmisweg 576 CH-4303 Kaiseraugst Switzerland; ^2^ Faculty of Medical and Life Sciences Institute of Precision Medicine Furtwangen University Jakob-Kienzle-Str. 17 Villingen‐Schwenningen 78054 Germany

**Keywords:** cosmetic, microorganisms, skin, skin care, skin microbiome

## Abstract

The human skin microbiome has recently become a focus for both the dermatological and cosmetic fields. Understanding the skin microbiota, that is the collection of vital microorganisms living on our skin, and how to maintain its delicate balance is an essential step to gain insight into the mechanisms responsible for healthy skin and its appearance. Imbalances in the skin microbiota composition (dysbiosis) are associated with several skin conditions, either pathological such as eczema, acne, allergies or dandruff or non‐pathological such as sensitive skin, irritated skin or dry skin. Therefore, the development of approaches which preserve or restore the natural, individual balance of the microbiota represents a novel target not only for dermatologists but also for skincare applications. This review gives an overview on the current knowledge on the skin microbiome, the currently available sampling and analysis techniques as well as a description of current approaches undertaken in the skincare segment to help restoring and balancing the structure and functionality of the skin microbiota.

## Graphical abstract

The human skin microbiome has recently become a focus for both the dermatological and cosmetic fields. Understanding the skin microbiota and how to maintain its delicate balance is an essential step to gain insight into the mechanisms responsible for healthy skin and its appearance. This review gives an overview on the current knowledge on the skin microbiome, the currently available sampling and analysis techniques as well as a description of current approaches undertaken in the skin care segment to help restoring and balancing the structure and functionality of the skin microbiota.

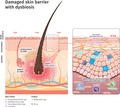

## Résumé

Le microbiome de la peau humaine est récemment devenu un centre d'intérêt pour les domaines dermatologique et cosmétique. Comprendre le microbiote cutané, à savoir la collection de microorganismes vitaux vivant sur notre peau, et comment maintenir son équilibre délicat est une étape essentielle pour mieux comprendre les mécanismes responsables d'une peau saine et son apparence. Les déséquilibres dans la composition microbiotique de la peau (dysbiose) sont associés à plusieurs affections cutanées, soit pathologiques comme l'eczéma, l'acné, les allergies ou les pellicules, soit non pathologiques comme la peau sensible, irritée ou sèche. Par conséquent, le développement d'approches qui préservent ou restaurent l'équilibre naturel et individuel du microbiote représente une nouvelle cible non seulement pour les dermatologues mais aussi pour les experts en cosmétiques. Cette revue donne un aperçu des connaissances actuelles sur le microbiome cutané, les techniques d'échantillonnage et d'analyse actuellement disponibles ainsi qu'une description des approches actuelles entreprises dans le segment des soins de la peau pour aider à restaurer et équilibrer la structure et la fonctionnalité du microbiote de la peau.

## The human cutaneous microbiome

The human skin is one of the largest organs of the body corresponding to a surface of 2 m^2^ which extends to approximately 25 m^2^ considering the plethora of hair follicles and sweat ducts 1, 2. This huge surface area is heterogeneous across the body, and it is continuously exposed to the external environment and has many vital functions. Skin acts as a physical, chemical, immunological, radiation and free radical barrier. Its main function is to maintain homeostasis by preventing water and extracellular fluid loss (permeability barrier), by keeping a constant body temperature through the perspiration process and by protecting the body from infection and toxic substances 3, 4. In addition, the skin harbours immune cells and is inhabited by billions of resident commensal microorganisms which constitute the so‐called skin microbiome (definitions associated with the microbiome are reported in Table 1) 5, 6, 7.

**Table 1:** Definitions associated with the microbiome research 8

Microbiota	The collection of vital microorganisms that live in or on a defined environment
Microbiome	The collection of all the microorganisms (bacteria, archaea, fungi, protozoa and viruses) that live in a particular environment or biome, their genomes and the surrounding environmental conditions including microbial metabolites (RNA, proteins, short‐chain fatty acids)
Dysbiosis	Imbalance of microbiome diversity and functionality
Prebiotics*	Non‐viable food components that confer a health benefit on the host associated with modulations of the microbiome structure and functionality
Probiotics*	Live microorganisms which when administered in adequate amounts confer a health benefit on the host 9
Post‐biotics*	Microbial metabolites and/or cell‐wall components released by probiotics
Metagenomics	Process used to characterize the metagenome, that is the collection of genes and genomes of all the microorganisms in a sample
Metabolomics	Large scale systematic identification and quantification of metabolic products (the metabolome) of a biological system (cell, tissue, organ, biological fluid or organism)
Metatranscriptomics	Gene expression profile of complex microbiomes
Metaproteomics	Large‐scale study of the proteome, that is, the protein expression profile of two or more species

* Definitions adopted from nutrition science. No clear definition yet for cosmetics.

Immediately after birth, the skin of newborns gets in contact with the maternal and post‐natal environment and becomes rapidly colonized by a diverse population of microorganisms such as bacteria, viruses and fungi 10, 11, 12, 13. The early‐life microbiota is thought to be of considerable importance since it stimulates the development of the immune system, its maturation and development of immune tolerance 14, 15. The majority of the microorganisms thriving on the human skin, defined as commensals or symbiotics, have been shown to provide protection against pathogens as well as to play an important role in the modulation of the host's cutaneous innate and adaptive immune system 16, 17. There are several lines of evidence indicating that antigens from skin commensal bacteria are detected by the host immune system even in the setting of an intact skin barrier. However, even though antigen‐specific T‐cell proliferation was detected in skin‐draining lymph nodes, no inflammatory reaction was observed suggesting a tolerogenic response to commensal microorganisms acquired during neonatal life 14. Besides that, skin‐resident bacteria produce acidic metabolites that together with the lactic acid present in our sweat and the free fatty acids coming from lipase‐mediated hydrolysis of phospholipids during cornification contribute to the low pH of the skin surface – the acid mantle−with which resident commensal bacteria can cope whereas many pathogens cannot 18, 19, 20, 21. Recent studies showed that the symbiotic relationship between some of the resident bacteria and the host is mutualistic since both profit from each other. The bacteria receive a place to live and feed in exchange of protecting the host from pathogens settling down on the skin. One example thereof is that certain strains of *Staphylococcus epidermidis* – one of the most abundant bacteria on the human skin *–* express a serine protease glutamyl endopeptidase (Esp) and produce antimicrobial peptides (bacteriocins), for example lantibiotics, which prevents colonization and biofilm formation by pathogenic *Staphylococcus aureus,* which is normally observed in chronic inflammatory skin diseases such as atopic dermatitis 22, 23, 24, 25, 26, 27, 28, 29.

## Microorganisms have topographical and environmental preferences

Generally, the skin microbiota exhibits a great variety of resident Gram‐positive bacteria such as *Staphylococcus, Cutibacterium* (formerly known as *Propionibacterium*) and *Corynebacterium* species whereas Gram‐negative bacteria are underrepresented and rather considered transient microorganisms. The most abundant fungi on human skin belong to the genus *Malassezia*, which predominates most body sites, aside from the feet where a higher fungal diversity is found 30, 31, 32, 33. Emerging evidence suggests that healthy‐looking human skin also harbours resident or transient viruses. As an example, cutaneous beta and gamma human papillomaviruses have been identified on the skin of most individuals 34, 35, 36. The heterogeneity of the skin surface depends on physiological characteristics (pH, temperature, sebum content and moisture (linked to the presence of sweat and sebaceous glands distributed across the skin)), topography (rough or smooth surface of the corneocytes) as well as on exogenous environmental factors – such as UV exposure, temperature and humidity – and it is reflected in a differential microbial colonization 30. In general, *Cutibacterium* is found thriving in sebaceous areas such as the forehead, whereas moist regions such as the navel or axilla have been found to be colonized preferentially by *Corynebacterium* and *Staphylococcus*. Dry areas like the volar forearm are characterized by the widest diversity of microorganisms, instead 37, 38, 39, 40. Moreover, the exact microbial community composition of the skin varies from individual to individual and interestingly remains quite stable over time 41. More specifically, even on a small area as the face, vast spatial and ethnic differences in skin conductance and transepidermal water loss (TEWL) were found and mapped, suggesting a diverse habitat and therefore a differential microbial colonization 42. Bouslimani and coworkers 43 created 3D topographical maps to visualize at a high spatial resolution both the chemical and microbial composition of the human skin surface. Interestingly, the study showed that the molecular composition of the skin varies across the body and differs among individuals even to a higher extent than the microbial community composition. These intra‐individual and inter‐individual diversities in microbiota/chemical composition represent a big challenge for skincare approaches. Microbial heterogeneity needs to be taken into account as there is an increasing awareness on the impact that cosmetic products have on the skin ecosystem 44. Recent evidences resulting from a study reported by Nakatsuji and coworkers 1 suggest that bacteria are not restricted to the skin surface, but bacterial metabolites, bacteria‐specific antigens as well as DNA and bacterial ribosomal RNA have been detected in deeper layers of the epidermis and even in the dermis and dermal adipose tissue, areas which were traditionally thought to be lacking a microbial community in absence of skin injury. The study did not directly provide any evidence that live bacteria thrive and inhabit the dermis as the approaches used were not able to discriminate between live or dead cells. However, even though the route of entry still has to be determined, it is assumed that live bacteria might be present in subepidermal compartments since bacterial RNA is rapidly removed after cell death and therefore a strong 16S rRNA hybridization signal would not be expected 45. Nevertheless, even though the study needs further support from more investigations, it represents an early evidence that a physical interaction between commensals, dermal cells and host immune system might occur.

## Bacterial strategies for skin colonization in health and disease

Bacteria colonizing the skin are subjected to a variety of mechanical and physico‐chemical stresses such as scraping or epithelial turnover as well as ultraviolet radiation 46, osmotic stress and shifts in pH. To withstand that, bacteria need to establish firm adhesion to the skin and therefore have developed a series of mechanisms which protect themselves and prevent their shedding from the skin surface. Initially, bacteria interact with the surface and adhere to it by non‐specific hydrophobic or electrostatic interactions. Subsequently, firm adhesion is achieved by bacterial expression of specific‐surface attached binding proteins (e.g. adhesins) interacting with human matrix proteins such as collagen, fibronectin, keratin, elastin and vitronectin. These bacterial surface proteins are collectively defined with the acronym MSCRAMMs (microbial surface components recognizing adhesive matrix molecules) 47, 48, 49. *S. epidermidis* and *S. aureus*, for example, have at least 18 and 29 genes, respectively, coding for surface binding proteins 50. If the skin conditions are permissive−for instance in case of skin disorders such as acne 51, 52 or atopic dermatitis 53 or in case of a wound 54 − after firm attachment bacteria start to proliferate to form entire colonies and stick to each other by producing extracellular matrix leading to the formation of bacterial biofilms 55, 56. However, even though many bacteria have virtually the ability to form biofilms, it is important to note that under normal physiological conditions the human skin does not allow the formation of biofilms as the requirements for their formation (e.g. moisture, pH, temperature) are missing. In a biofilm bacteria enable multicellular functions and metabolic changes and benefit from advantageous survival mechanisms allowing them to survive in hostile environments which often translate into virulence, resistance to antibiotics and pathogenesis 57. In such aggregation state, bacteria can either physically interact with each other but also communicate by releasing, sensing and responding to small signal molecules, an activity called quorum sensing 58, 59. Such way of communication is paramount for the synchronization of activities and responses to changes within the microbial community, allowing unicellular organisms as bacteria to act and behave like multicellular organisms. For example, in the event of a skin wound or a lesion, bacteria colonize the wounded tissue and further proliferate being in a favourable environment. While growing, bacteria produce communication molecules responsible for quorum sensing called autoinducers. Through these molecules, bacteria can synchronize their behaviour to secrete virulence factors and produce biofilms leading to, for example reduced efficacy of antibiotic treatment. If the autoinducers are degraded or inhibited (quorum quenching) bacteria cannot synchronize anymore and therefore remain harmless. The wound remains colonized but without signs of infection. Several plant‐derived molecules have been shown to have the potential to interfere with quorum sensing 60, 61, 62. Quorum quenching approaches as well as biofilm dispersal strategies 63 – which trigger the release of biofilm‐associated microbes into their more vulnerable, planktonic state−are appealing and may have important implications in many different medical fields including dermatology as they do not directly impact on bacterial survival, with a consequent low selection pressure therefore avoiding the occurrence of resistance 64, 65.

## Currently available sampling methods to analyse the skin microbiota

In most cases, the analysis of the human skin microbiota requires that the microorganisms living on our skin are retrieved and their nucleic acids sequenced. The sampling methods which are currently used to harvest the skin microbiota are skin swabbing, tape‐stripping and punch biopsy 66. Skin swabbing is one of the most commonly used methods as it is quick, simple, non‐invasive and suitable for large‐scale skin sampling. It is performed using a sterile swab which is pre‐moistened in 0.9% sodium chloride with 0.1% Tween‐20 and rubbed on the skin surface 31, 34, 35, 67. However, the collection efficacy may be significantly influenced by the conditions of swabbing such as the number of strokes and the pressure applied. A study carried out by Van Horn K. and coworkers 67 has compared three different swab transport systems in terms of release and recovery capabilities with either aerobic and anaerobic bacteria. It has been shown that the material of which the swab is made (e.g. flocked swab or rayon‐tipped swab) has an impact in the collection yield as well as in the direct release and recovery of the isolated microorganisms. The ESwab™ (flocked swab) appeared to be able to release more efficiently (10‐fold more) the microorganisms compared to the other two rayon‐tipped swabs 67. The tape‐stripping method involves the use of adhesive tape to collect the skin microbiota. This technique has been used in several studies as an alternative to skin swabbing 68, 69. In contrast to the swabbing technique, the tape‐stripping method allows to ‘peel off’ bacteria from the stratum corneum as well as deeper layers. A recent study by Ogai and coworkers 70 showed that next generation sequencing analysis of microbial samples collected with the tape‐stripping method revealed a slightly higher abundance of *Cutibacterium spp.,* which are known anaerobic bacteria, compared to the ones collected using the swabbing method. This might suggest that the tape‐stripping technique allows for a deeper sampling of the stratum corneum reaching the anaerobic portions of the skin appendages. Overall, the study concluded that both techniques are equivalent in terms of skin microbiome analysis. Interestingly, the tape‐stripping method collected more viable bacteria than the swabbing method 70. Another stripping method includes the use of cyanoacrylate glue 71. It represents a low‐cost method to sample a continuous sheet of stratum corneum and horny follicular casts in a minimally invasive way. However, there are two main limitations. This method is conveniently used on glabrous area of the body, as the sampling from a hairy area is typically painful because of the pulling of the hair, and furthermore, the sampling quality is inadequate because of the partial contact of the glue with the stratum corneum. A second limitation results from the natural strong intercorneocyte cohesion on the palms and soles. This force is commonly stronger than the glue bond and impairs the collection of a uniform layer of corneocytes. Punch biopsies, instead, are invasive but offer the best representation of skin microbiota 35 as they allow for the collection of full‐thickness skin specimens comprehensive of superficial (mostly aerobic bacteria) as well as deep skin flora (anaerobic bacteria).

## Planning a human skin microbiome study

Once understood the advantages and disadvantages of each sampling method, researchers which intend to investigate the skin microbiome need to pay special attention to the planning of clinical studies. As for any other study, statistical power, for instance, is of considerable importance and must be taken into consideration at the start of a microbiome study 72, 73. In view of the great individuality and variability of the skin microbiota, an adequate number of subjects must be recruited and it is reasonable that each proband serves as his/her control. Besides that, since the human skin microbiota is sensitive to both exogenous and endogenous factors, these must be taken into account as well. For instance, sex, drug use, antibiotic treatments, age, diet, geographical origin, season (winter, summer), and even pet ownership, all have been showed to impact the function and composition of the skin microbiota 74. Once the pool of volunteers has been selected, the sampling of skin microbiota is of considerable importance. The area to be sampled has to be clearly defined and has to be large enough because of the fact that the skin microbiota is so diverse even between nearby areas. Furthermore, the sampling preferably is performed by the same trained person in order to reduce inter‐operator variabilities. As previously mentioned, the number of strokes, the pressure applied and even the type of swab used directly impact the quality of the biomass sampled and therefore need to be standardized. Once collected, the samples should ideally be processed immediately. If this is not possible, they can be stored at −80°C but should still be processed in a relatively short time frame. A recent publication by Klymiuk and coworkers 75 analysed the long‐term (up to 1 year) storage effect at −80°C on the results of microbial composition (16S rRNA seq). Even though the study was performed only with samples collected from 8 volunteers, it interestingly showed significant changes in the relative abundance and ratios of some of the dominant phyla and genera of each skin location at the different timepoints. The DNA, RNA, proteins and metabolites extraction and analysis of the skin microbiome samples require special attention as well, since technical biases can easily be introduced at this stage. The following section focuses on this topic.

## Approaches to characterize and study the skin microbiome

Historically, the first approaches aimed at understanding the composition of the human skin microbiota were culture‐based, including cultivation of live colonies on agar plates. However, later it became evident that only the minority of microorganisms are able to thrive in isolation. Culture‐based studies are limited as only the most abundant and rapidly growing bacteria are selected by the culture conditions resulting in an underestimation of microbial community diversity 34, 76, 77. Furthermore, anaerobic bacteria thriving in the anoxic conditions of hair follicles or sebaceous glands are quite problematic to isolate and cultivate using standard routine approaches as they have fastidious growth requirements 78. However, the cultivation method is still widely used as standard technique in clinical laboratory tests which require living microbial cultures, such as antibiotic‐susceptibility and virulence testing or genome sequencing. Therefore, both cultivation approaches and exhaustive metatechnological analyses are essential for microbiome studies 79. In 2009, the National Institute of Health funded the Human Microbiome Project (HMP) with the aim to characterize the microbial communities present at specific body sites and to further the understanding on how the microbiome impacts human health and disease 80. In recent years, next generation genomic technologies have revolutionized research on the human cutaneous microbiome. Advances in genomic technology enabled the development of new culture‐independent approaches based on DNA sequencing 81, 82. Bacterial communities are most commonly classified and identified by the sequence of their small subunit 16S ribosomal RNA (rRNA) gene as it is ubiquitous and highly conserved between different species of bacteria 83. The advantage of targeting the 16S rRNA gene is that it is comprised of conserved and variable regions. Universal primers can be designed to target the portion of conserved regions which are immediately adjacent to the variable ones. Through PCR analysis, the variable regions can be amplified allowing for the determination of the various species. The 16S rRNA gene is nevertheless exclusive to prokaryotes (bacteria and archaea); thus, it does not provide any information on the other components of the skin microbiota, such as fungi for whose the 18S rRNA gene or the internal transcribed spacer (ITS) region might be targeted in a similar way 84. In contrast to 16S rRNA sequencing, the whole genome sequencing (WGS), defined also as shotgun metagenomic sequencing, surveys the entire genetic material in a sample without amplification of specific target regions. It works by partially digesting the genome into small overlapping fragments which are sequenced and fragments that overlap are matched together. Additionally, WGS provides the possibility to extract the potential of each genome. The combination of the two different approaches allow for the identification of the bacterial community composition at genus‐ and sometimes at species‐level (16S rRNA gene) and for the taxonomic classification at species‐ and even sometimes at strain‐level (Shotgun metagenomic analysis). However, considering the amount of resources and costs which have to be incurred into when performing these methods, the choice of using one or the other approach has to be made during the experimental design considering the final purpose of the investigation. For instance, when aiming at differentiating commensals from pathogens, species‐ and, even better, strain‐level resolution is strongly recommended in order to have a clear identification of the bacteria 85. Both 16S rRNA gene sequencing and shotgun metagenomic sequencing require attention to contaminations. Contaminating DNA coming from DNA extraction reagents, kits (the so‐called kitome) 86 and water has been reported in several studies 87, 88, 89, 90, 91. Compared to the human intestinal tract, skin samples contain only very low microbial biomass; hence, environmental contaminants may easily lead to false‐positives, and therefore, the sequencing of adequate negative controls is highly recommended 66, 81, 82, 92, 93. The qualitative analysis provided by the 16S rRNA sequencing or by the WGS can be flanked by qPCR which allows to quantify the microorganisms present in the sample. The two analyses give an overview of who is present and to what extent. However, a standard qPCR does not discriminate between live and dead bacteria. Viability PCR with propidium monoazide (PMA) helps to overcome this limitation by allowing the detection of the living bacteria only. PMA is a membrane impermeable photoreactive DNA‐binding dye which labels dead bacteria and inhibit their DNA amplification. Although many studies so far were DNA‐based and have focused on the structure (species inventory) of the skin microbiota, more studies are needed that address microbiota functionality (RNA, proteins, metabolites). Therefore, in addition to the abovementioned metagenomic approaches (taxonomical profile), metatranscriptomics as well as metaproteomics and metabolomics are increasingly being adopted to understand the dynamics and functionality of the cutaneous microbiota 94, 95. Metatranscriptomics allows for the identification of genes which are actually transcribed, proteomics and metabolomics approaches identify and quantify proteins as well as metabolites which are released into the environment, respectively. In the absence of budget constraints, a polyphasic approach which combines metagenomic, metatranscriptomic, metaproteomics and metabolomics including cultivation would definitely provide an exhaustive overview and would help to elucidate not only structural but also functional changes in the skin microbiome that accompany a pathological state.

## How should a healthy microbiota look like?

The skin in its entirety can be defined as a complex and dynamic ecosystem. Besides the physical barrier provided by the stratum corneum, the skin‐resident microbiota guarantees protection and a biological barrier by competing with pathogens and by communicating closely with cells and components of the immune system to modulate either the local and systemic immune responses 17, 96. Therefore, it is reasonable to consider the cutaneous microbiota an essential player for the maintenance of a healthy skin. J. M. Crowther nicely suggests that the stratum corneum should be no longer considered as a simple layer of dead cells (the corneocytes) but instead a layer supporting a complex ecosystem, a stratum ecologica 97. Skin barrier structure and function is essential to human health. It is well known that there is a balanced interplay between the host and the bacterial populations which is continuously exposed to host, intrinsic factors as well as environmental, extrinsic factors 38, 39, 98, 99. A sustained imbalance in the microbial community composition, defined as dysbiosis, characterizes several skin disorders such as eczema, allergies, dandruff or acne 3.

**Figure 1:**
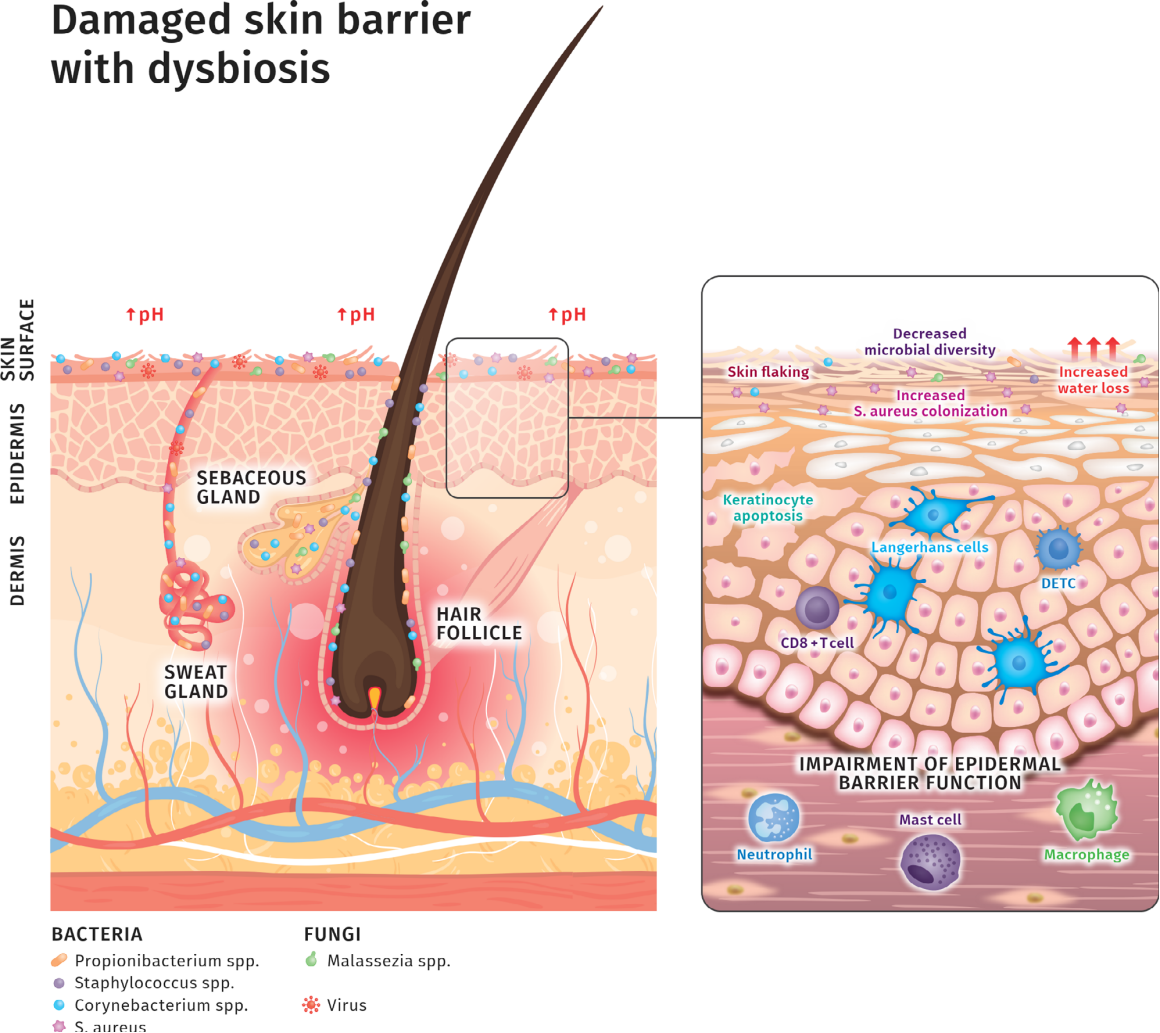
Damaged skin barrier with dysbiosis. The state of dysbiosis (imbalance) is typical of some chronic inflammatory skin diseases such as atopic dermatitis, psoriasis, rosacea or acne. The skin barrier is weakened, skin pH raises and water loss dramatically increases. Skin flaking and keratinocytes apoptosis also occur. All these changes are accompanied by a sustained inflammation with involvement of immune cells such as Langerhans cells, dendritic epidermal T cells (DETC), neutrophils, macrophages and mast cells. Interestingly, it becomes more and more evident that the microbiota composition is affected by these biochemical and biophysical changes resulting in a decreased microbial diversity and increased colonization by pathogenic bacteria, such as *Staphylococcus aureus* which is known to form biofilms in skin disorders such as atopic dermatitis. However, we are still far away from determining whether microbial dysbiosis is the cause or the consequence of such skin biophysical modifications 7, 104.

Nevertheless, because of the huge inter‐ and intra‐individual variability in skin microbiota composition depending on the skin site it might result difficult to define how a healthy microbiota should look like and moreover, its role in skin health and disease is far from being fully understood. In recent years, research has focused on identifying changes in the microbiota occurring in skin disease. An investigation carried out in 2013 by Fitz‐Gibbon S. and coworkers 100 highlighted that, rather than the entire species, certain *Cutibacterium acnes* strains have been shown to be responsible for the occurrence of acne although other strains were enriched in healthy skin. Several lines of evidence suggest microbial diversity being a requisite for skin health as it characterizes many skin disorders 101, 102, 103. For instance, *Staphylococcus aureus* colonization in atopic dermatitis patients was predominant in about 90% of the cases and this was associated with a loss of skin microbiota diversity suggesting that dysbiosis with increased *S.aureus* colonization is an important factor exacerbating the pathogenesis of atopic dermatitis (Fig. 1) 7, 101.

It is globally accepted that commensal bacteria might convert themselves to pathogenic in particular conditions. *Staphylococcus epidermidis* is widely classified as a bacterium beneficial to skin health. It is known to inhibit *Staphylococcus aureus* biofilm formation by production of the serine protease glutamyl endopeptidase (Esp) and also stimulates keratinocytes to produce antimicrobial peptides resulting in *S.aureus* killing 26, 28, 105. However, despite these multiple beneficial functions, *S.epidermidis* is still classified as one of the most important pathogens in nosocomial infections associated with catheters and other medical implants. On the other hand, a recent study showed for the first time that a strain of commensal *S.aureus* isolated from human perinasal skin revealed the ability to produce short‐chain fatty acids−known for their bactericidal activity – as products of glycerol fermentation and to elicit both innate and adaptive immunity responses against infection by methicillin‐resistant *Staphylococcus aureus* 106. Most of the time, there is a healthy and concerted balance between our skin and the microorganisms living on it. However, environmental stresses and other factors can cause a shift of our skin microorganisms from commensal to pathogenic, resulting in inflammation, itching, scaling and other clinical signs of imbalance between our skin and the microbiota. Considering the high inter‐individual variability in the microbiota composition and the many factors affecting it, with the current knowledge we cannot fully answer the question yet: How should a healthy microbiota look like? What appears to be important is the maintenance of a balanced and biodiverse ecosystem. However, increased bacterial diversity must be cautiously intended as beneficial, since it does not always correspond to healthy skin status. For instance, elderly skin has a higher bacterial diversity and this is most likely due to several factors related to skin ageing such as, among others, reduced skin cells renewal, and old skin being more permissive to bacterial colonization compared to young skin 107.

## Present and future perspectives in the skincare industry

The microbiome represents a still largely unexplored but rapidly emerging field in the personal care industry. Conventional beauty and skincare products contain synthetic chemicals and antimicrobial preservatives which might impact, for better or for worse, the delicate balance of the cutaneous microbiota. However, despite the widespread use of skincare and hygiene products, their effects on the structure and functionality of the skin microbiota are still unknown and should be investigated in much more detail. A recent study carried out by Bouslimani and coworkers evaluated the influence of personal care products on the skin in terms of microbial and molecular composition 44. The key findings were the following: (1) Molecules associated with personal skin and hygiene products last on the skin for weeks after their first use despite regular showering. (2) Molecular and bacterial diversity were altered following beauty products usage. Another study sought to assess the effects of cosmetic underarm products on the axillary microbiota 108. When deodorants or antiperspirants were used in a consistent way, the ecological balance was shifted in a stable manner leading to the establishment of a new balance 108, 109. Likewise, another study reported that the use of makeup on the forehead seemed to cause important structural community alterations 110. Nevertheless, the understanding of these shifts, their influences on skin health and disease susceptibility needs to be further investigated.

Following the exploding interest on the skin microbiome topic, an increasing number of companies have focused on this market opportunity and started to develop ‘biome friendly’ skincare products. The current approaches undertaken are focused on either preventing the removal of ‘good’ bacteria by adapting product formulation or to restore ripped off bacteria−for example after showering – with products added with prebiotics or probiotics. Particularly, *Staphylococcus epidermidis* caught the attention as several beneficial effects are attributed to this bacterium: The ability to inhibit *S. aureus* colonization by maintaining a low pH and by secreting antimicrobial substances as well as the improved skin moisture provided by its metabolic products such as glycerine and organic acids 111. With the aim of boosting the level of these beneficial effects Nodake and coworkers 112, in a randomized, placebo‐controlled pilot study, isolated autologous skin *S. epidermidis* from subject‐specific skin microbiota, expanded it by culturing, incorporated in a gel after lyophilization and applied twice a week to the subject face to increase colonization. Interestingly, the study revealed that topical application of *S. epidermidis*−which led to a significant increase in the relative count of *S. epidermidis* over the 4 weeks of treatment−greatly influenced the retention of the skin moisture, reduced water evaporation and increased relative lipid content with the latter being most likely the reason why a moisture retention effect was observed. Interestingly, an increased amount of glycerine and lactic acid was observed on the skin of the treated group which would support the improved moisture retention observed. Unfortunately, the study did not consider evaluating the topical application of dead *S. epidermidis* as further control. Beside *S. epidermidis*, also ammonia‐oxidizing bacteria (AOB) have been taken into consideration as important players in the conversion of irritating components of the sweat such as ammonia and urea into by‐products (nitrite and nitric oxide) which are supposed to bring benefits to the skin. AOB are hypothesized by AOBiome scientists to be historical commensal colonizers of our skin before we started to wash them away with our improved and modern hygiene practices. Therefore, they hypothesize that the reintroduction of AOB on the cutaneous ecosystem may have a positive impact on skin health. Several clinical trials have been carried out to assess the efficacy of a single strain of AOB, *Nitrosomonas eutropha*, for instance in subjects with atopic dermatitis or even with seasonal allergic rhinitis (https://clinicaltrials.gov/ct2/results?term=AOBiome). However, there are no published results yet from these trials.

Despite the efforts to show the benefits in adding living microorganisms to the skin, there are several concerns which need to be addressed. As previously mentioned, many typical skin bacteria are potentially pathogenic (risk group 2), which clearly hampers their use as probiotics. Interestingly, many probiotics currently used for cosmetic purposes (lactic acid bacteria, AOB) are classified as risk group 1 and are not typical members of the human skin microbiota, but often well‐known probiotics from the intestinal tract. Nevertheless, topical application of such selected bacteria is thought to interfere with the colonization by other, potentially pathogenic, bacterial strains through competitive inhibition of binding sites, a mechanism defined as bacterial interference 113.

**Figure 2:**
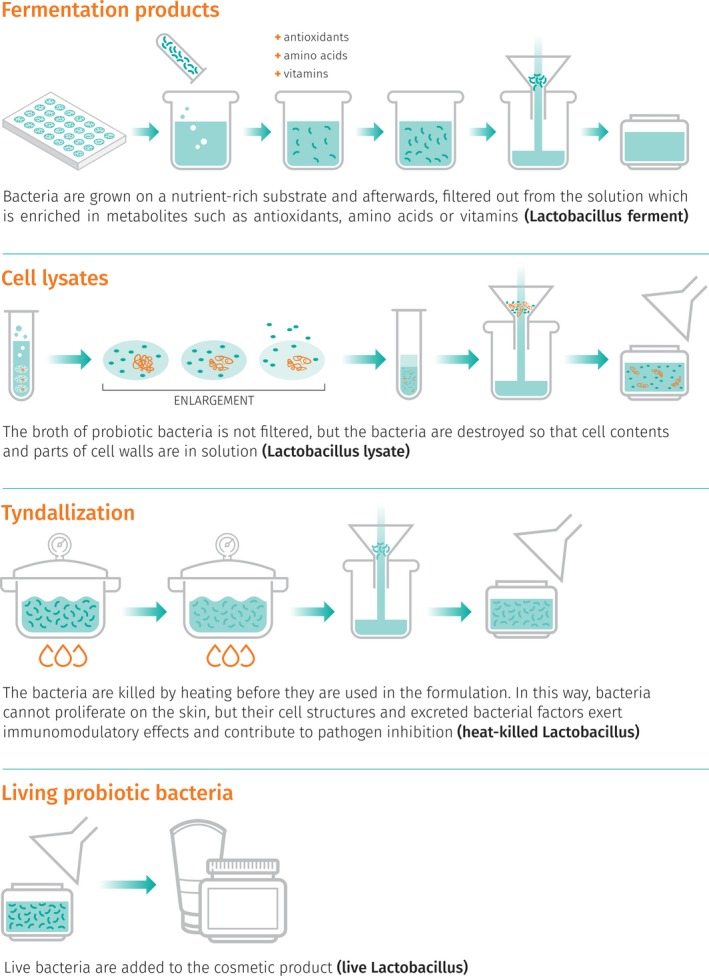
‘Probiotic ingredients’ in cosmetics (INCI names are given in brackets).

There are, however, already several studies, performed in animal models, which have shown that the ingestion of probiotics led to a dramatical improvement of both mucosal and skin health supporting the theory of the brain‐gut‐skin connection and indicating potential health benefits 114, 115, 116, 117. Another concern which needs attention is the safety of cosmetics products containing probiotics. Even though, currently there is no regulatory definition of probiotics in cosmetic products and FDA has no premarket authority, companies or individuals manufacturing or marketing cosmetics must ensure the safety of their products. Furthermore, there are some technical barriers which need to be overcome to be able to introduce live bacteria into conventional skincare products while ensuring a reasonable shelf life. Most cosmetic products contain a large amount of water and preservatives are used to prevent bacterial growth and spoilage. Therefore, generally, cosmetic products do not contain bacteria. To easily circumvent this technical limitation, some companies marketed products containing non‐viable bacteria, products of bacterial fermentation or cell lysates (which do not require a real change in the preservative system) as ‘probiotic’ or ‘probiotic ingredients’. A clear nomenclature and coherent definitions, however, are still missing in cosmetics which may lead to confusion among consumers. So far, the terminology used to define the presence of bacteria or their extracts in cosmetic products is borrowed from nutrition science; however, the term ‘probiotic’ is enriched with a broader meaning and often includes ingredients that are not directly living bacteria, but which have been obtained by means of probiotic bacteria (Fig. 2).

Nevertheless, if planning to use live bacteria in a cosmetic formulation the following concerns remain to be addressed: How can the beneficial live bacteria be kept alive in cosmetic products? How can cosmetics containing live probiotics meet the regulations on microbial contamination limits? How can product safety be assessed and assured? Currently, only few products containing live bacteria (mostly Lactobacilli) are available on the market. Although the precise mechanisms by which lactic acid bacteria may improve skin health is still unknown, several studies focused on showing beneficial effects on skin health 118, 119, 120. One of the few products available on the market contains live Lactobacilli and is formulated as facial serum; thus, it does not contain water. Furthermore, the bacteria are encapsulated. It is claimed that bacteria are activated as soon as they come into contact with skin moisture, after mechanical rupture of the capsules. However, no literature supporting this theory is available yet. Another biotech company proposes micro‐encapsulated freeze‐dried Lactobacilli in an oil‐in‐water cream 121. Last but not least another approach, inspired by the success of faecal microbiota transplantation for the treatment of *Clostridium difficile* infection 122, suggests the transplantation of bacterial communities from one individual to another as well as the topical application of selected beneficial bacterial strains to modulate and rebalance the microbiota composition when pathological dysbiosis occurs, for example for the treatment of acne vulgaris or atopic dermatitis 123, 124, 125. Given the promising results, it is reasonable to assume that, in the near future, we will see more and more cosmetic products embracing the new philosophy of taking care (protect or balance) of the skin microbiota. It is furthermore rather likely that we will not just see a single bacterial strain adopted as a ‘treatment’ but rather a ‘healthy’ bacterial community including some additional fungal elements. These would more realistically reflect the overall microbiota composition of a balanced and healthy skin. In addition, we might foresee the advent of personalized skincare approaches as well as dermatological treatments where consumers/patients will have the possibility to access products or medicaments which are tailored for their specific skin microbial needs.

## Conclusions

In summary, this review provides an overview of the current knowledge and approaches undertaken to better characterize the skin microbiome as well as the future perspectives for the skincare industry. The technical advances in DNA extraction of low biomass samples and sequencing techniques have been crucial for the current, even though still limited, understanding of the structure of the skin microbiota. More investigations are needed, and research should focus not only on the structure but also on the functionality of the skin microbiota 126 in order to provide answers to the following questions: What role do microorganisms have in our skin and how do they contribute to the maintenance of skin homeostasis? Is dysbiosis the cause or the consequence of a pathological status? Can pathological strains be replaced with non‐pathological ones and ameliorate disease or skin disorders? How is the microbiota involved in sensitive, irritated and dry skin? In the future, the current analyses of microbiome sequencing data should be supported with metabolomic, metaproteomic and metatranscriptomic profiling combined with skin biophysical measurements to correlate microbiome structure/function data with the skin barrier status and to provide a better picture of the healthy or disease‐associated microbiota.

## Conflicts of interests and disclosures

RS, MG, RV and RC are employees of DSM Nutritional Products Ltd. ME is a consultant to DSM Nutritional Products Ltd.
